# Supplementary material for: Mannan-Binding Lectin Is Associated with Inflammation and Kidney Damage in a Mouse Model of Type 2 Diabetes
Source: Int J Mol Sci. 2024 Jun 29;25(13):7204. doi: 10.3390/ijms25137204 (PMC11241296; doi:10.3390/ijms25137204)
Supplement: Supplementary file 1 [file ijms-25-07204-s001.zip › ijms-2959472-supplementary.pdf]

Table S1: Biomarkers of the lectin pathway, nephropathy, and inflammation in 5-week-old mice

Biomarker levels 5-week-old mice	Wild type-mice (n=6)	OB-mice (n=6)	P-value
MBL-A (µg/ml)	47.7 CI(37.8 ; 57.6)	97.0 CI(73.8 ; 120.2)	<0.001
MBL-C (µg/ml)	76.0 CI(66.8 ; 85.2)	177.3 CI(117.7 ; 236.8)	0.007
C3-fragments (mU/ml)	73.6 IQR(9.4)	44.4 IQR(74.3)	0.393
Total-C3 (µg/ml)	1747.9 CI(1520.97 ; 2008.65)	1637.5 CI(1358.6 ; 1973.6)	0.489
Cystatin C (ng/ml)	463.1 CI(373.0 ; 553.1)	501.3 CI(428.2 ; 574.4)	0.417
SAA-3 (ng/ml)	35.6 IQR(13.4)	141.6 IQR(36.2)	0.002
